# Evaluation of the Solidification of Radioactive Wastes Using Blast Furnace Slag as a Solidifying Agent

**DOI:** 10.3390/ma16196462

**Published:** 2023-09-28

**Authors:** Ji-Hun Jeon, Jong-Hwan Lee, Woo-Chun Lee, Sang-Woo Lee, Soon-Oh Kim

**Affiliations:** 1 Department of Geology and Research Institute of Natural Science (RINS), Gyeongsang National University (GNU), Jinju 52828, Republic of Korea; 2 HS Environmental Technology Research Center, Hosung Inc., Jinju 52818, Republic of Korea

**Keywords:** concrete waste, blast furnace slag, decommissioned nuclear-power facilities, solidification, radioactive waste

## Abstract

The decommissioning process of nuclear power facilities renders hundreds of thousands of tons of various types of waste. Of these different waste types, the amount of concrete waste (CW) varies greatly depending on the type of facility, operating history, and regulation standards. From the previous decommissioning projects, CW was estimated to comprise 60–80 wt.% of the total weight of radioactive wastes. This represents a significant technical challenge to any decommissioning project. Furthermore, the disposal costs for the generated concrete wastes are a substantial part of the total budget for any decommissioning project. Thus, the development of technologies effective for the reduction and recycling of CW has become an urgent agenda globally. Blast furnace slag (BFS) is an industrial byproduct containing a sufficient amount (higher than 30%) of CaO and it can be used as a substitute for ordinary Portland cement (OPC). However, there have been few studies on the application of BFS for the treatment of radioactive waste from decommissioning processes. This study was conducted to evaluate the performance of the solidification agent using ground granulated BFS (SABFS) to pack radioactive wastes, such as the coarse aggregates of CW (CACW), waste soil (WS), and metal waste (MW). The analytical results indicated that the CaO content of the ground granulated BFS was 36.8% and it was confirmed that calcium silicate hydrate (CSH) could be activated as the precursor of the hydration reactions. In addition, the optimum water-to-binder ratio was determined to be 0.25 and Ca(OH)_2_ and CaSO_4_ were found to be the most effective alkaline and sulfate activators for improving the compressive strength of the SABFS. The maximum packing capacities of the SABFS were determined to be 9 and 13 wt.% for WC and WM, respectively, when the content of CW was fixed at 50 wt.%. The results of the leaching tests using SABFS containing radioactive wastes contaminated with Co, Cs, and Sr indicated that their leachability indices met the acceptance level for disposal. Consequently, the SABFS can be used as a solidifying agent for the safe disposal of radioactive waste.

## 1. Introduction

As of August 2023, according to the statistics served by the Power Reactor Information System (PRIS) of the International Atomic Energy Agency (IAEA), globally, 410 nuclear power plants are in operation, 209 are end-of-life plants, and only 21 were decommissioned entirely [1]. Of those in operation, 68% and 24.5% are ancient facilities with average life spans longer than 30 and 40 years, respectively, and worldwide decommissioning processes are expected to increase gradually [2,3]. When decommissioning nuclear facilities, hundreds of thousands of tons of various wastes, such as concrete, metal, soil, and liquid waste, are generated; it has become a pending global issue to acquire technologies related to volume reduction, solidification and stabilization, and recycling [4,5]. Generally, there are no general methods to estimate the amount of waste generated from the decommissioning of nuclear power plants because it is very different depending on the type of facility, operating history, and regulatory standards. However, Song et al. (2015) estimated the average amount of waste generated from decommissioning one nuclear plant to be about 5000 tons [6]. In addition, regarding the amounts of generated waste according to countries, they expected 5300 tons for Germany, 15,400 tons for France, and 9500 tons for Japan [6]. The European Commission (EC) expects that approximately 500 million tons of concrete waste (CW) will be generated from nuclear decommissioning facilities in Europe until 2060 [7]. It is likely that solid waste constitutes approximately 70–80% of the nuclear decommissioning waste and that 70% of solid waste can be classified as CW.

The legal definition and classification of radioactive wastes according to the level of radioactivity are different between countries, and here, the Korean regulation is introduced [8]. The ultralow level is defined as radioactivity over the standard for the self-disposal and smaller than 100 times the standard for its self-disposal given in the Supporting Information (Table S1). In addition, the low level of radioactivity is over 100 times the standard for self-disposal and smaller than the standard for the radioactivity of low-level radioactive wastes, as shown in Table S2. In terms of the intermediate-level radioactive wastes, they have radioactivity over the standard for the radioactivity of low-level radioactive wastes (Table S2). Finally, the high-level radioactive waste is the waste that does not belong to any radioactive wastes defined above. The high-level radioactive waste has a heat production of 2 kW/m^3^ and radioactivity over 4000 Bq/g. The proportion of CW that is radioactive is less than 5% and more than 95% of CW is considered as ultralow-level radioactive [9]. At present, the cost of the disposal of this ultralow-level radioactive CW is cut down through the use of landfills, incineration, and recycling [10,11,12]. Technologies to minimize the amount of nuclear decommissioning wastes and to recycle them have been actively developed in advanced countries such as France, Japan, Belgium, Germany, Spain, the UK, and the USA. In addition, France has completed experiments and reached the demonstration stage [13,14]. 

Blast furnace slag (BFS) is an industrial byproduct that is generated from steel mills. Its CaO content is sufficiently high (≥30%) enough to arouse hydration reactions that are similar to ordinary Portland cement (OPC) [15,16]. There have been studies on manufacturing high-strength concrete using materials as a substitute for OPC, such as fly ash and micro-silica [17,18]. Calcium silicate hydrate (CSH) and ettringite (Ca_6_Al_2_(SO_4_)_3_(OH)_12_∙26H_2_O) are known to be primary products generated from the hydration reactions of OPC and CSH is the main product of BFS hydration [19]. Due to the hydraulic property of BFS, it can be used as a substitute or replacement for OPC. For this reason, there have been numerous studies conducted to reduce the emission of carbon dioxide (CO_2_) by decreasing the usage of OPC with the substitution of ground granulated blast furnace slag (GGBFS) [20,21,22]. GGBFS is a kind of industrial waste and its disposal requires cost. Therefore, the recycling of GGBFS can reduce such costs. Moreover, carbon reduction is additionally achieved due to the use of GGBFS as a substitute for OPC and this is invaluable economically and environmentally. GGBFS has various merits: it improves the compressive strength of solidification materials as a result of generating large amounts of hydration products such as CSH due to its high CaO content and it also shows high acid resistance, excellent corrosion resistivity, and large long-term strength. Despite its strengths, there have been few studies undertaken to evaluate its applicability for the safe disposal of radioactive waste that is generated from the decommissioning processes. As BFS shows potential hydraulicity, the process of removing its surface film is required so as to give rise to the hydration reactions caused by OPC [23,24,25].

Recently, geopolymer concretes have been greatly spotlighted due to their economical effectiveness, wide availability, large-scale applicability, and environmental friendliness because of the raw materials used, such as municipal wastes and industrial byproducts, which are inexpensive and readily available, making geopolymers very cheap [26,27,28]. Notably, Shilar et al. (2023) conducted a comprehensive literature survey to investigate the effects of various binders in geopolymerization, such as fly ash, GGBFS, clay, metakaolin, and red mud [26]. In addition, Han et al. (2023) confirmed the feasibility of the utilization of municipal solid waste incineration fly ash with coal fly ash/metakaolin for geopolymer composites preparation [27]. Moreover, this study focused on the solidification of radioactive wastes using GGBFS as a substitute for OPC; the process evaluated in the study seems identical to geopolymerization because geopolymers are made from raw materials and can make concrete-like materials. Therefore, the solidification process using GGBFS considered in this study can be identically understood as geopolymerization. The reason why the term solidification was used instead of geopolymerization was that the study focused on the immobilization of radioactive wastes through the solidification process using GGBFS to dispose of them safely rather than the manufacture of geopolymer concrete.

This study was conducted to evaluate the manufacturing process of a solidification agent using GGBFS (SABFS) for the safe packing of radioactive wastes, such as the coarse aggregates of CW (CACW), waste soil (WS), and metal wastes (MW) that originate from the decommissioning process of nuclear facilities. The specific objectives were to (1) determine the optimum mixing ratio of GGBFS, (2) elucidate the manifestation mechanism of compressive strength, (3) assess the maximum packing capacity of SABFS for radioactive wastes based on the disposal acceptance criterion of the compressive strength, and (4) evaluate the leachability of SABFS according to the types of radioactive waste and radionuclides contained.

## 2. Materials and Methods

### 2.1. Materials

The BFS used in this study was a byproduct generated from the process of producing pig iron at the Pohang Iron and Steel Company (Pohang, Republic of Korea) and the GGBFS was obtained by crushing and grinding it into fine powders of sizes smaller than 0.38 μm to improve hydration reactions. Three types of hydroxides, NaOH, KOH, and Ca(OH)_2_, were used as alkaline activators to enhance GGBFS hydration [29,30,31]. For the experiments conducted to investigate the fundamental properties of SABFS and to elucidate the manifestation mechanism of its compressive strength, CACW (larger than 5 mm in size) were used as the target packing waste because they are known to be the most common waste generated from the decommissioning process [32]. Stepwise experiments were conducted using CACW, WS, and MW to measure the maximum capacity of the SABFS to solidify radioactive waste. As it was impossible to bring actual radioactive wastes into the laboratory for experiments, CW was simulated by producing the concrete. Generally, the concrete used for the construction of nuclear power facilities is manufactured following the American Concrete Institute (ACI) 301 and 304 guidelines and the mixing ratios (wt.%) for OPC, coarse, and fine aggregates are 10–15, 40–45, and 40–50, respectively [33,34]. In this study, the CW was manufactured by blending OPC with fine and coarse aggregates with the ratios (wt.%) of 20.8, 38.9, and 40.3; moreover, water was added at a water-to- binder (W/B) ratio of 0.38. After a 28-day curing period of CW, before experiments, CACW was obtained by crushing it to a size that was larger than 5 mm. The WS was simulated using soil samples taken from a site near the Wolsong Nuclear Power Plant located in Gyeongju City, which is an operating nuclear power plant. The particle size of soil samples used as WS was smaller than 75 μm. In contrast, MW was simulated with a stainless-steel plate (SUS 304) because it is known to be a common waste generated from the decommissioning process [35]. It was cut into 5 × 5 × 1 mm pieces before the experiments.

The maximum capacity of the SABFS was determined based on whether its compressive strength, which contained radioactive waste, satisfied the acceptance level (3.44 MPa). The criterion on the compressive strength for disposal used in this study was proposed by the Korea Radioactive Waste Agency (KORAD). In addition, as it was impossible to obtain actual radioactive solid wastes; the target radioactive wastes-CACW, WS, and MW were considered solid-phase wastes with low-level radioactivity, and legally, they should be solidified to be disposed of because their radioactivity levels exceeded 100 times the standard for the self-disposal (Table S2). First, a series of experiments were conducted to investigate the maximum capacity of SABFS in terms of containing CACW; this was conducted because they account for the most considerable portion of CW that originates from decommissioned nuclear facilities [36]. Based on the results, it was confirmed that the compressive strength of the SABFS was acceptable when the CACW content was 50 wt.%. Subsequently, the maximum capacity of the SABFS to contain WS and MS was determined using a fixed ratio (50 wt.%) of CACW. In addition, the leachability of radionuclides after solidification was evaluated using WS and MS. As it was impossible to acquire actual radioactive wastes and bring them into the laboratory, these wastes were artificially spiked with non-radionuclides, such as Co, Cs, and Sr, which were used to simulate radionuclides. Meanwhile, it is reported that regardless of radioactivity, nuclides exhibit similar chemical behavior; non-radionuclides were used instead of radionuclides [36]. Leaching tests were conducted according to the international method coded by the American Nuclear Society (ANS) 16.1 and leachability was calculated using Equation (1) [7]:

(1) $$L_{i}=\frac{1}{10}\sum_{1}^{10}\left[\log\left(\frac{\beta }{Di}\right)\right]$$

*L_i_* = the leachability of the *i*-th radionuclide;

*β* = a constant, with a value of 1.0 cn^3^/s;

*D_i_* = the effective diffusion coefficient of the *i*-th radionuclide.

### 2.2. Mixing Proportions and Curing

The primary objectives of the study were to (1) determine the optimum blending ratio of SABFS, (2) elucidate the manifestation mechanisms of its compressive strength, (3) determine the maximum capacity of SABFS for the containment of radioactive wastes, and (4) measure the leachability of the radionuclides included in radioactive wastes.

First of all, the optimum mixing ratio for the manufacture of SABFS was determined through experiments with different conditions, such as W/B ratios; the type of alkaline and sulfate activators; and curing methods. Each experimental condition is presented in Table 1. Additionally, four curing methods, such as air drying, water, atmospheric steam, and high-pressure and high-temperature curing, were tested to compare the compressive strengths between the curing methods. Table 2 shows the detailed experimental conditions.

Next, we investigated the manifestation mechanism of the compressive strength mechanism by adding the representative hydration products, such as CSH and ettringite, which are known to improve the compressive strength of cement. Using low-graded limestone containing SiO_2_ (13.8 wt.%), Al_2_O_3_ (3.0 wt.%), and CaO (42.8 wt.%), CSH was made by the method suggested by Moon et al. (2016) [37]. Ettringite was manufactured using C_3_A (3CaO∙Al_2_O_3_, tricalcium aluminate) and gypsum (CaSO_4_∙2H_2_O) following the method proposed by Shim et al. (2012) [38]. In addition, we evaluated which of these were more effective in enhancing the strength. The experimental conditions are presented in Table 3. 

Thirdly, a series of experiments were conducted to determine the maximum capacity of the SABFS in terms of containing radioactive wastes, such as CACW, WS, and MW. The detailed experimental conditions are given in Table 4. 

Finally, to evaluate the leachability of each type of radioactive waste and radionuclides, three types of radioactive waste were used after they were artificially spiked with Co, Cs, and Sr. The experimental conditions are listed in Table 5. The leachability tests were conducted using ANS 16.1 after a 28-day curing period and the variation in the compressibility of SABFS was compared before and after the leaching tests. The SABFS was manufactured according to the different mixing ratios shown in Table 3, Table 4 and Table 5 and the manufacturing procedure was as follows: (1) all the materials were mixed in a dry state for approximately 30 s, (2) water was gradually added and the mixture was stirred for approximately 30 s, (3) the target waste to be contained was added and mixed for approximately 30 s, and finally (4) the mixtures were introduced into molds with a size of 27 × 27 × 54 mm, which were then cured using different methods after compaction. 

### 2.3. Measurement of Compressive Strength

According to different conditions, the compressive strength of each SABFS specimen manufactured was evaluated to verify whether it met the acceptance criterion (3.44 MPa) required by the disposal sites. Following ASTM C39 and KS F2405 [39,40,41], the compressive strength of the SABFS was measured four times every week (7, 14, 21, and 28 days) during the curing period. The compression was conducted in a displacement-controlled mode at a speed of 10 mm/min with a digital laboratory CBR test apparatus (DA-551, Dong Ah Machine, Seoul, Republic of Korea) while using cubes with dimensions of 27 × 27 × 27 mm. Furthermore, the compressive strength was measured in quintuples using five specimens that were fabricated under the same conditions because the compressive strength appeared to be different between the samples. Accordingly, the average value of the five measurements was computed to improve the reliability and significance of the results.

### 2.4. Instrumental Analyses

First, the chemical compositions of the CACW and GGBFS, which were used to manufacture SABFS, were investigated using X-ray fluorescence (XRF) (S8 TIGER, Bruker AXS, Billerica, MA, USA). After the last measurement of the compressive strength at the end of the 28-day curing period, the specimens were demolded to observe their mineralogical composition and microstructure. The demolded specimens were first crushed and ground using a ball mill until all the powders were smaller than 74 µm; their mineralogical composition was measured using an X-ray diffractometer (XRD) (Siemens D5005, Cu-Kα, 40 kV, 35 mA, step size: 0.02 Deg, Time/step: 5 s). Finally, we prepared the polished sections with the SABFS specimens that were cut after the measurement of their compressive strength. Then, their microstructure and the products of the hydration reaction were observed using a Raman spectrometer (Ramantouch, Nanophoton, Osaka, Japan) and a low-accelerating voltage field emission scanning electron microscope (JSM-7900F, JEOL, Peabody, MA, USA). The analyses of the Raman spectrometer were conducted using a laser with a wavelength of 785 nm, a 20-s duration, a laser spot of 50 μm, and a laser output of 11 mW. As the peaks of the Raman spectra were different depending on the locations, we measured more than 20 points to improve the analytical reliability, and the results served as average values. 

## 3. Results and Discussion

### 3.1. Properties of Materials

The chemical compositions of the CACW and GGBFS analyzed by the XRF are given in Table 6. The CaO content of the GGBFS was measured to be 36.8% and CSH was expected to form as the precursor of the hydration reactions occurring in cementitious materials. This indicated that it is likely to manufacture a solidifying agent when using GGBFS to pack radioactive waste.

**Figure 1:**
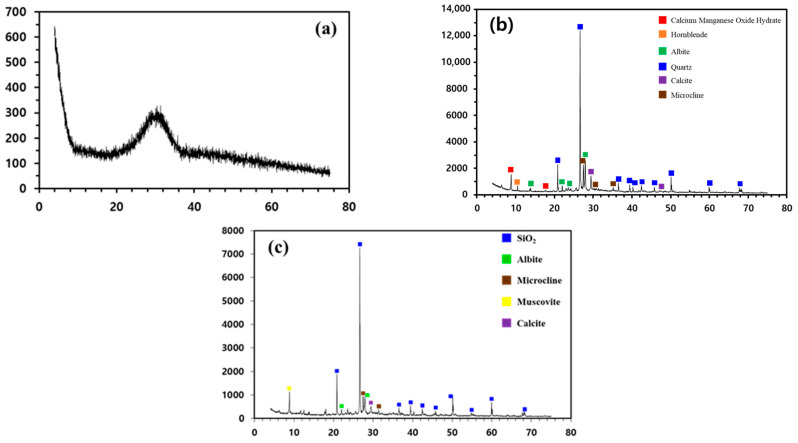
The X-ray diffractograms of the following: (**a**) ground granulated blast furnace slag (GGBFS), (**b**) coarse aggregates of concrete waste (CACW), and (**c**) waste soil (WS).

The XRD results for the mineralogical compositions of GGBFS, CACW, and WS are shown in Figure 1. The poor degree of crystallinity of the GGBFS was confirmed by its XRD diffractograms. In the case of CACW, the peaks of quartz were overwhelming and those of albite and muscovite were identified. Meanwhile, the intensities of the calcite peaks were fragile despite the relatively higher CaO content. The XRD results of WS were particularly similar to those of CACW and the quartz peaks were remarkable, followed by those of albite and microcline.

### 3.2. Optimum Mixing Ratio

#### 3.2.1. Water-to-Binder Ratio

**Figure 2:**
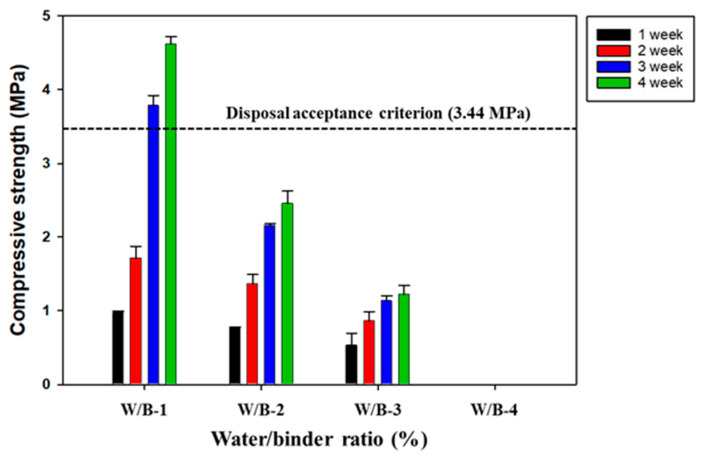
The effect of W/B ratios on the compressive strength of the solidification agent using blast furnace slag (SABFS). Refer to the detailed experimental conditions in Table 1.

The water-to-binder (W/B) ratio is one of the most crucial factors affecting the strength and durability of SABFS and it should be determined by considering its workability [42]. Vasovic et al. (2021) suggested that it is necessary to determine the optimal W/B ratio to improve compressive strength and that the compressive strength increases with a decreasing W/B ratio [42]. In addition, the range of the W/B ratio for the concrete of high-strength is known to be 0.25–0.3 [43,44]. The optimal W/B ratio was determined when the compressive strength exhibited the highest value with the consideration of workability. For this reason, a series of experiments were conducted to optimize the W/B ratio (Table 1) and the results are given in Figure 2. The compressive strength of SABFS was measured to be 4.6 MPa, which met the disposal criterion (3.44 MPa), and this was recorded when the W/B ratio was 0.25 after the 28-day curing period. In the case of a 0.3 W/B ratio, the compressive strength of SABFS was decreased by up to half of the 0.25 W/B ratio. When the W/B ratio was 0.4, it was impossible to measure the compressive strength because the SABFS was not hardened even after a 28-day curing period. These results can be attributed to the fact that the water was likely to exceed the quantity needed for the hydration reaction and the fact that the surplus water was likely to form capillary pores rather than facilitate hydration reactions [43]. With the consideration of the disposal criterion of compressive strength and workability, a W/B ratio of 0.25 seemed the optimum level. 

#### 3.2.2. Type of Alkaline and Sulfate Activators

**Figure 3:**
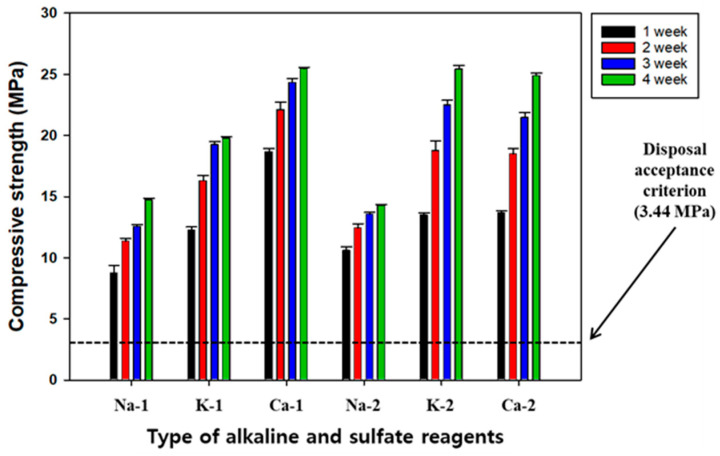
The compressive strength of the solidification agent using blast furnace slag (SABFS) according to the types of alkaline and sulfate reagents. Refer to the detailed experimental conditions in Table 1.

It is necessary to introduce alkaline and sulfate activators to promote the hydration reaction when stimulating the surface of GGBFS; this is because it exhibits potential hydraulicity as its chemical components do not dissolve in contact with water. Alkaline activators dissolve the chemical components of GGBFS and generate hydration products such as CSH, ultimately improving the compressive strength of SABFS. Park et al. (2013) suggested that the GGBFS with the potential hydraulicity can be stimulated by the alkaline activators, resulting in the destruction of its surface and the transformation of its structure to gel-type [45]. As a result, its compressive strength can be improved due to the formation of hydration products, such as CSH and ettringite, similar to the OPC. NaOH, KOH, and Ca(OH)_2_ are the most frequently used alkaline activators; however, there have been few studies on their performances in stimulating surfaces, producing hydration products, such as CSH, and improving the compressive strength of cementitious materials. For this reason, the effects of different alkaline and sulfate activators on the compressive strength of SABFS were investigated according to the experimental conditions shown in Table 1, of which the results are presented in Figure 3. 

Prior to the main experiments, preliminary tests were conducted to determine their dosages and the results indicated that the highest compressive strength of SABFS was obtained at a combination of 35 wt.% GGBFS, 7.5 wt.% alkaline, and 2.5 wt.% sulfate activators. In addition, this ratio was used in the main experiments to select the more effective types of each activator. After the 28-day curing period, the compressive strength increased in the following order: Ca(OH)_2_ > KOH > NaOH. On the other hand, it was not likely that sulfate activators affected the compressive strength of SABFS. The compressive strengths of all cases tested were measured to be 10 MPa, which was recorded immediately after a curing period of only seven days and they all met the disposal criteria. In the case of Ca(OH)_2_, the compressive strength of the SABFS after the 28-day curing was 27 MPa, which represented the highest among the cases tested. Park et al. (2013) reported that NaOH and KOH acted as effective catalysts for stimulating the surface of GGBFS but Ca(OH)_2_ disrupted the formation of hydration products, such as CSH, as a result of the consumption of Si by Ca [45]. In contrast, Park and Choi (2013) reported that Ca(OH)_2_ could improve the compressive strength because the formation of hydration products such as CSH and calcium aluminate hydrates (CAH) was enhanced due to the efficient interaction of Ca with SiO_2_ and Al_2_O_3_ [46]. In addition, Shim (2010) reported that the alkaline and sulfate materials containing Ca, such as Ca(OH)_2_ and CaSO_4_, worked as activators, and simultaneously, they acted as primary reactants consumed for the hydration reaction of GGBFS [47]. Furthermore, they also suggested that unlike Ca(OH)_2_, NaOH and KOH only acted as catalysts without taking part in generating reaction products [47]. The results of this study indicate that when Ca(OH)_2_ was used as an alkaline activator, the compressive strength was improved due to the formation of CSH via the interaction of Ca with SiO_2_. Additional efforts are needed to scrutinize the possibility of decreasing the compressive strength caused by the disruption of surplus Na and K in the hydration reaction when NaOH and KOH are used as alkaline activators. 

### 3.3. Effect of Curing Methods on the Compressive Strength of SABFS

**Figure 4:**
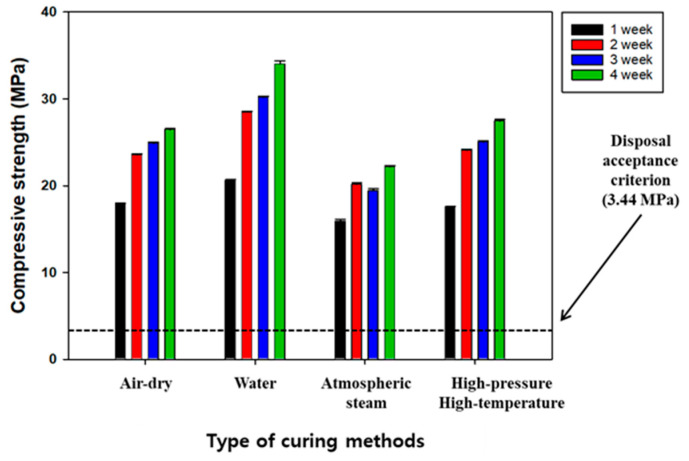
The effect of curing methods on the compressive strength of the solidification agent using blast furnace slag (SABFS). After a 28-day curing period, samples for each curing method were demolded in air to measure their compressive strengths. Refer to the detailed experimental conditions in Table 1 and Table 2.

Compared with OPC-based concrete, the SABFS exhibited a much lower compressive strength but blending it with OPC was required to improve its compressive strength. However, the purpose of the study was to maximize the content of GGBFS in manufacturing SABFS and the experiments were conducted to find the curing methods that are effective in enhancing the compressive strength of SABFS. The most frequently used curing methods, such as air drying, water, atmospheric steam, and high-pressure and high-temperature curing, were investigated by considering their effects on the compressive strength of SABFS, the experimental conditions of which are presented in Table 1 and Table 2. As shown in Figure 4, the results showed that the methods with the best increasing effect on the compressive strengths were in the following order: water > high-pressure and high-temperature > air-dry > atmospheric steam. In addition, all curing methods satisfied the disposal criterion. Furthermore, even though the compressive strength of the SABFS using GGBFS as a substitute for OPC was inferior to that of OPC (30–40 MPa), it was estimated to be higher than 20 MPa in all the conditions, and particularly, it was measured about 35 MPa in the case of water curing. The compressive strength of the SABFS was relatively lower in the air-dry, atmospheric steam, and high-pressure and high-temperature curing methods and it was attributed to insufficient hydration reactions due to the evaporation of the water. On the other hand, the water curing method resulted in a higher compressive strength due to the gradual formation of hydration products, such as CSH and ettringite (Ca_6_Al_2_(SO_4_)_3_(OH)_12_∙26H_2_O), which occurred as a result of the continuous interaction of water and hydration precursors, such as β-C_2_S. Even though the experimental results showed that the best curing method was water curing, it is recommended that the air-drying curing method be considered due to its suitability regarding the disposal standard, cost-effectiveness, and convenience.

### 3.4. Manifestation Mechanism of the Compressive Strength

**Figure 5:**
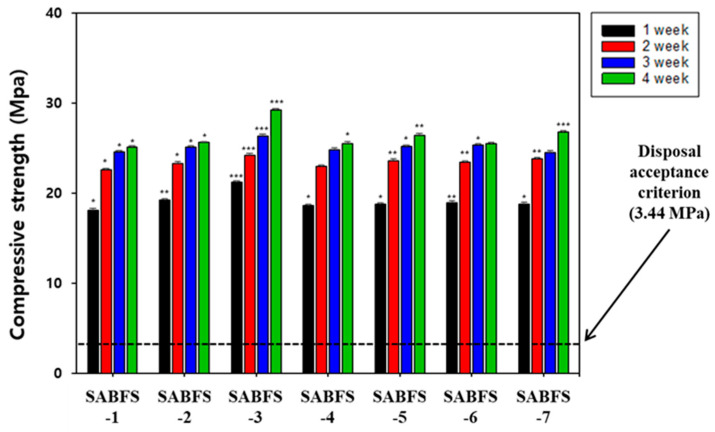
The effect of the type of hydration products, such as calcium silicate hydrate (CSH) and ettringite, on the compressive strength of the solidification agent using blast furnace slag (SABFS). Refer to the detailed experimental conditions in Table 3. * Significance (*p*-value) ≤ 0.05, ** Significance (*p*-value) ≤ 0.01, and *** Significance (*p*-value) ≤ 0.001.

The representative hydration products of OPC are known to be CSH and ettringite, while CSH is a primary product for slag-type materials [48,49]. In addition, calcium hydroxide (CH) can be produced in both cases. CSH is reported to have a colloidal size and significantly affects the strength of cement [50]. Meanwhile, the ettringite of needle-like crystals is the first product of the OPC hydration reactions and improves the initial strength of cement [51]. This ettringite improved the compressive strength during the initial stage of hydration reactions as a result of filling the pores formed between paste and aggregates. The ettringite is finally transformed into a monosulfate [Ca_4_Al_2_O_6_(SO_4_)∙12H_2_O], which has sheet-like hexagonal crystals [52]. On the contrary, if the amount of calcium sulfate and aluminate is relatively more remarkable, then the etrringite cannot be transformed into a monosulfate. In the case of an identical amount between both materials, ettringite and monosulfate coexist. However, there has been insufficient effort to elucidate the effect of hydration products, such as ettringite and CSH, on the compressive strength of cement. Therefore, in this study, CSH and ettringite were intentionally added to evaluate their impact on the compressive strength of SABFS and the specific conditions of these experiments are given in Table 3. As shown in Figure 5, the specimens, following the introduction of hydration products, exhibited higher compressive strengths than those without them. In particular, it was confirmed that the compressive strengths satisfied the disposal criterion after a curing period of only seven days with the addition of hydration products. Furthermore, CSH was observed to be more effective at increasing compressive strength than ettringite. In addition, the statistical significance of the results was confirmed, as shown in Figure 5. Comparing the *p*-values between the results, the cases with the addition of CSH exhibited relatively smaller values (higher significances) than those with ettringite and without both materials, indicating that CSH was likely to be more effective than ettringite.

**Figure 6:**
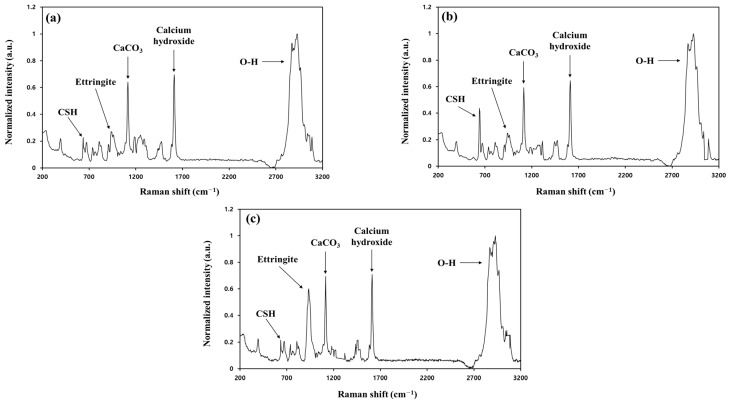
The Raman spectrometer results on the solidification agent using blast furnace slag (SABFS) after the addition of hydration products, such as calcium silicate hydrate (CSH) and ettringite: (**a**) without the addition of CSH and ettringite, (**b**) with the addition of CSH (5 wt.%), and (**c**) with the addition of ettringite (5 wt.%).

**Figure 7:**
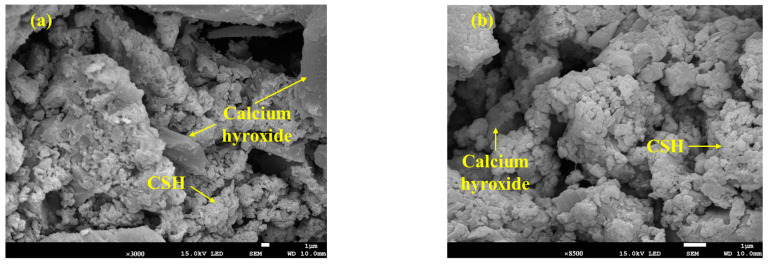

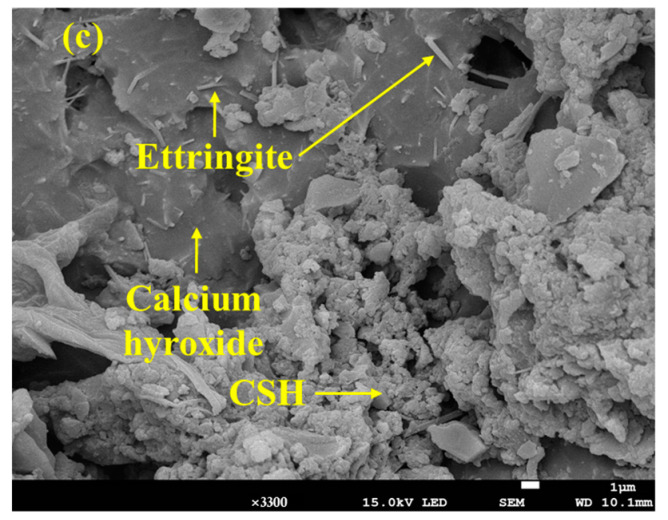
The effect of the addition of hydration products, such as calcium silicate hydrate (CSH) and ettringite, on the microstructure of the solidification agent using blast furnace slag (SABFS): (**a**) without the addition of CSH and ettringite, (**b**) with the addition of CSH (5 wt.%), and (**c**) with the addition of ettringite (5 wt.%).

Analyses via Raman spectrometry and SEM were conducted to investigate the manifestation mechanism of the compressive strength of both materials. Three different types of SSBFS specimens were prepared for the analyses: (1) with neither substance, (2) with the addition of 5% CSH, and (3) with the addition of 5% ettringite. The results are shown in Figure 6 and Figure 7. Based on the results of the Raman analyses, the peaks of CSH and ettringite were identified at 650 and 960 m^−1^, respectively, and those of CH and CaCO_3_ were also observed (Figure 6). The intensity of the CSH peaks was twice as strong in the case of adding CSH as those in the case without CSH, thus indicating that CSH considerably affected the compressive strength of SABFS. In addition, the SEM analyses were conducted to investigate the microstructure of SABFS, the results of which are given in Figure 7. In SABFS without the presence of either material, we observed CSHs above the sheet-like CH, as well as some filled pores (Figure 7a). On the other hand, in the SABFS with the addition of CSH, larger clusters of small CSH particles were identified above the sheet-like CH and they seemed to fill the pores (Figure 7b). Finally, when ettringite was added, gel-shaped CSHs and ettringites with a size of 1–5 μm were observed above the sheet-like CH, which filled the pores (Figure 7c). The pore volume increased in the following order: none of the materials > ettringite > CSH. However, the compressive strength values showed the opposite tendency. Consequently, the effect of CSH on compressive strength was found to be significant because of the blocking of the pore spaces.

### 3.5. Simulation of the Immobilization of Radioactive Wastes Using SABFS

When nuclear power facilities are decommissioned, a considerable amount of solid waste, such as CW, WS, MS, and resins, is generated. The compressive strength of materials solidifying those wastes is influenced differently by the type of waste contained. Thus, experiments were conducted to simulate the solidification of these wastes when using SABFS and the changing tendency of the compressive strength was observed. In addition, the maximum packing capacity of the SABFS for radioactive waste was determined. As mentioned in Section 2.1, because the CACW is likely to account for the most considerable portion of the solid waste generated from the decommissioning process, the maximum possible amount of it that can be contained in SABFS was investigated first. The results showed that 50 wt.% was the maximum proportion of CACW that can be packed in SABFS. Then, the maximum solidifying capacities of the SABFS for WS and MW were evaluated using a fixed ratio (50 wt.%) of CACW. In addition, the leachability of radionuclides was indirectly evaluated by using non-radionuclides such as Co, Cs, and Sr, which were artificially spiked into the waste. For the experiments on the leachability of WS, the maximum adsorption capacities of the non-radionuclides were determined in advance to select the appropriate spiking level and the maximum adsorbed concentrations (MAC) of Co, Cs, and Sr were estimated as 23,700, 10,000, and 17,200 mg/kg, respectively. Based on the results, they were spiked at three different levels: (1) 10% lower than MAC, (2) equal to MAC, and (3) 10% higher than MAC. In the case of MW, it was impossible to determine the MAC of each nuclide, and it was sufficiently soaked for 24 h in a solution that contained them at a concentration of 50,000 mg/L before the leachability tests. The experiments were conducted following ANS 16.1 and the leaching indices of each nuclide were estimated after the 90-day leaching tests. The suitability of each waste type was determined by comparing the leaching indices with an acceptance level of six. In addition, the compressive strength of the SABFS containing each waste was reanalyzed after the leaching tests to confirm the stability of the SABFS.

#### 3.5.1. Maximum Packing Capacity of SABFS for the Radioactive Wastes 

**Figure 8:**
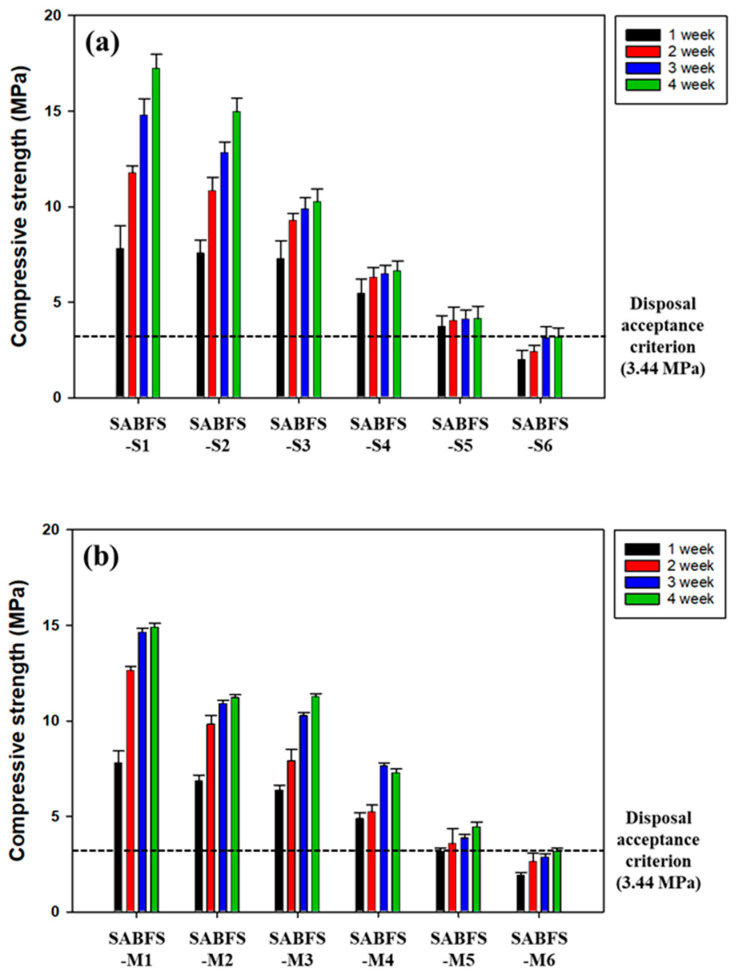
The maximum packing capacities of the solidification agent using blast furnace slag (SABFS) for the simulated radioactive wastes: (**a**) coarse aggregate of concrete waste (CACW) + waste soil (WS) scenario and (**b**) coarse aggregate of concrete waste (CACW) + metal waste (MS) scenario. Refer to the detailed experimental conditions in Table 4.

The maximum packing capacities for the WS and MW are shown in Figure 8 and the corresponding experimental conditions are given in Table 4. The compressive strength of the SABFS containing CACW (50 wt.%) and WS (9 wt.%) was investigated to meet the disposal criterion after a 28-day curing period. In contrast, 50 wt.% CACW and 13 wt.% MW exhibited compressive strengths that satisfied the standard. In the case of MW, the maximum packing capacity of SABFS was anticipated to be much larger due to its relatively larger particle size but it appeared to be somewhat smaller than expected; this resulted from the formation of interstitial pores due to decreasing GGBFS content and bonding strength. Comparing the compressive strengths between WS and MW, the former exhibited much smaller values than the latter in the case of the equivalent amount of both wastes packed, e.g., 9 and 10 wt.% in SABFS-S5 and S6 for WS and SABFS-M1and M2, as shown in Table 4 and Figure 8. This was attributed to the fact that WS could not act as an aggregate in SABFS due to its small particle size.

#### 3.5.2. Leachability of Radionuclides Contained in the Radioactive Wastes

**Figure 9:**
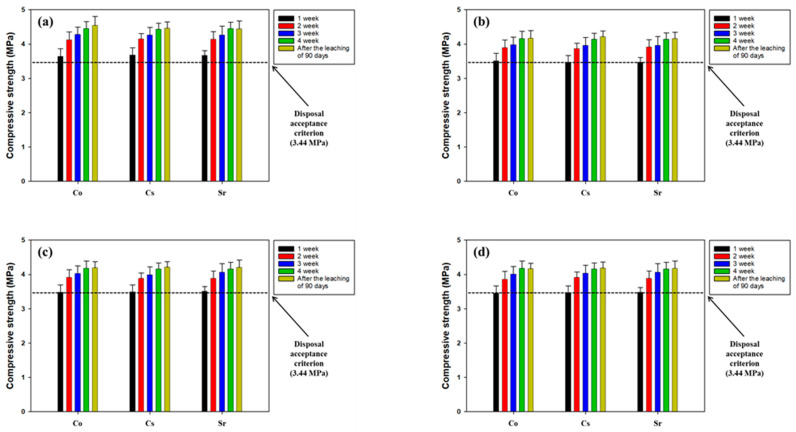
The comparison of the compressive strengths of the solidification agent using blast furnace slag (SABFS) according to the types of radioactive wastes and radionuclides: (**a**) metal waste (MW) soaked in the solutions of Co, Cs, and Sr scenario, (**b**) waste soil (WS) spiked by Co scenario, (**c**) waste soil (WS) spiked by Cs scenario, and (**d**) waste soil (WS) spiked by Sr scenario. Refer to the detailed experimental conditions in Table 5.

Based on the results of the maximum packing capacity of the SABFS for the simulated radioactive wastes, the amounts of each waste used for the leachability tests were 50 wt.% for CACW, 9 wt.% for WS, and 13 wt.% for MW. The other specific experimental conditions are presented in Table 5. The leaching indices of each nuclide are given in Table 7 and all values met the acceptance level (6) for disposal. In the case of WS, even though its leaching indices satisfied the criterion, they decreased with increasing amounts of spiking, which was attributed to the fact that some nuclides could be leached out through pores of SABFS because the sites for immobilization decreased with increasing loadings. A comparison of the compressive strength of SABFS during the curing period and after the leaching tests is shown in Figure 9. In all the scenarios of the different wastes, the compressive strengths of SABFS were found to meet the standard after a 28-day curing period, regardless of the type of nuclide. In addition, it was confirmed that the compressive strength of SABFS did not change after the 90-day leaching tests in all the scenarios. Consequently, the results indicated that the solidifying agent using GGBFS can be used to safely pack a considerable amount (more than 50 wt.%) of contaminated radioactive waste with different radionuclides.

## 4. Conclusions

This study was conducted to evaluate the applicability of ground granulated blast rnace slag—a byproduct that is generated in the process of producing pig iron so as to manufacture a solidifying agent for the safe packing of radioactive wastes, such as coarse aggregates of concrete waste, waste soil, and metal wastes that originate from the decommissioning process of nuclear facilities which are considered the most common and above-medium-level radioactive wastes. Specifically, the optimum mixing ratio of materials, the effect of curing methods on compressive strength, the manifestation mechanism of compressive strength, the maximum packing capacity for radioactive wastes, and the leachability of radionuclides were assessed. 

The XRF results on the chemical composition of ground granulated blast furnace slag confirmed its potential as a substitute for ordinary Portland cement because it exhibited a significant CaO content (approximately 37%) and it was expected to produce calcium silicate hydrate, which is the precursor of hydration reactions.

The optimum ratio of water to the binder was measured to be 0.25. In addition, it was confirmed that the compressive strength of a solidifying agent was the highest when using 7.5 wt.% Ca(OH)_2_ and 2.5 wt.% CaSO_4_ as the alkaline and sulfate activators, respectively. After 28-day curing, all the curing methods satisfied the disposal criterion of compressive strength and the water curing method showed the highest compressive strength (35 MPa) among the curing methods tested. Based on the experimental results that elucidated the manifestation mechanism of the compressive strength, CSH was the most effective in improving the compressive strength of SRC over ettringite. The maximum packing capacities of the solidifying agent were achieved when 50 wt.% coarse aggregates of concrete waste were contained with 9 wt.% waste soil or 50 wt.% coarse aggregates of concrete waste with 13 wt.% metal waste. Finally, the results of the leaching tests on the simulated radionuclides, such as Co, Cs, and Sr, met the leachability standards for disposal. Furthermore, the compressive strength of the solidifying agent met its criterion after 90-day leaching tests. Consequently, the results suggest that GGBFS can be used as an alternative to ordinary Portland cement to manufacture a solidifying agent for the safe disposal of radioactive wastes.

More efforts will be needed to make up for the limitations of the study, such as elucidating the difference in performance and economy between GGBFS and OPC and quantitative evaluation of impacts of porosity, microstructures, hydration degree, and workability. After that, the study should be expanded for practical implementation through applications of the engineering scale. In addition, the extension to immobilize and fix liquid-phase radioactive wastes should be evaluated for practical implementation.

## Figures and Tables

**Table 1:** Experimental conditions for determining the optimum blending ratio of materials (unit: weight %).

	Exp. No.	Binder						Simulated Radioactive Waste (Concrete Waste ≥ 5 mm)	Water/ Binder Ratio	Curing Condition
		GGBFS (≤0.38 μm)	Alkali Activator			Sulfate Activator				
			NaOH	KOH	Ca(OH)_2_	Na_2_SO_4_	CaSO_4_			
Water/ binder ratio	W/B-1	30	15	-	-	5	-	50	0.25	Air-dry
	W/B-2	30	15	-	-	5	-	50	0.3	
	W/B-3	30	15	-	-	5	-	50	0.35	
	W/B-4	30	15	-	-	5	-	50	0.4	
Ratio of activator	Na-1	40	7.5	-	-	2.5	-	50	0.25	Air-dry
	K-1	40	-	7.5	-	2.5	-	50	0.25	
	Ca-1	40	-	-	7.5	2.5	-	50	0.25	
	Na-2	40	7.5	-	-	-	2.5	50	0.25	
	K-2	40	-	7.5	-	-	2.5	50	0.25	
	Ca-2	40	-	-	7.5	-	2.5	50	0.25	
Curing method	Air-dry	40	-	-	7.5	-	2.5	50	0.25	Air-dry
	Water	40	-	-	7.5	-	2.5	50	0.25	Water
	Atmospheric steam	40	-	-	7.5	-	2.5	50	0.25	Atmospheric steam
	High pressure and high temperature	40	-	-	7.5	-	2.5	50	0.25	High pressure and high temperature

**Table 2:** Conditions of different curing methods.

Method	In the Mold with a Size of 27 × 27 × 54 mm		Demolded	
	Condition	Period (Hour)	Condition	Period (Day)
Air drying	In a room environment (20 °C and 50% relative humidity)	24	In a room environment (20 °C and 50% relative humidity)	28
Water	In a room environment (20 °C and 50% relative humidity)	24	In a water tank (20 ± 2 °C)	28
Atmospheric steam	In a room environment (20 °C and 50% relative humidity)	2	After natural cooling, in a room environment (20 °C and 50% relative humidity)	28
	Gradually increased at a rate of 15 °C/h	4		
	Maintained at 65 °C	6		
High pressure and high temperature	In a room environment (20 °C and 50% relative humidity)	4	In a room environment (20 °C and 50% relative humidity)	28
	10 atm, gradually increased at a rate of 90 °C/h	2		
	10 atm, maintained at 180 °C	7		
	Under the pressure of 10 atm, gradually decreased at a rate of 90 °C/h until reached 20 °C	2		

**Table 3:** Experimental conditions for elucidating the manifestation mechanisms of compressive strength (unit: weight %).

Exp. No.	Binder					Simulated Radioactive Waste (CACW ≥ 5 mm)	Water/ Binder Ratio	Curing Condition
	GGBFS (≤0.38 μm)	CSH	Ettringite	Alkali Activator Ca(OH)_2_	Sulfate Activator CaSO_4_			
SABFS-1	40	-	-	7.5	2.5	50	0.25	Air-dry
SABFS-2	37.5	2.5	-	7.5	2.5	50	0.25	
SABFS-3	35	5	-	7.5	2.5	50	0.25	
SABFS-4	37.5	-	2.5	7.5	2.5	50	0.25	
SABFS-5	35	-	5	7.5	2.5	50	0.25	
SABFS-6	37.5	1.25	1.25	7.5	2.5	50	0.25	
SABFS-7	35	2.5	2.5	7.5	2.5	50	0.25	

**Table 4:** Experimental conditions for determining the maximum capacity of the solidification agent using blast furnace slag (SABFS) to contain radioactive wastes (unit: weight %).

Exp. No.	Binder			Simulated Radioactive Waste			Water/ Binder Ratio	Curing Condition
	GGBFS (≤0.38 μm)	Alkali Activator Ca(OH)_2_	Sulfate Activator Ca_2_SO_4_	CACW (≥5 mm)	WS (≤0.75 mm)	MW		
SABFS-S1	35	7.5	2.5	50	5	-	0.25	Air-dry
SABFS-S2	34	7.5	2.5	50	6	-	0.25	
SABFS-S3	33	7.5	2.5	50	7	-	0.25	
SABFS-S4	32	7.5	2.5	50	8	-	0.25	
SABFS-S5	31	7.5	2.5	50	9	-	0.25	
SABFS-S6	30	7.5	2.5	50	10		0.25	
SABFS-M1	31	7.5	2.5	50	-	9	0.25	Air-dry
SABFS-M2	30	7.5	2.5	50	-	10	0.25	
SABFS-M3	29	7.5	2.5	50	-	11	0.25	
SABFS-M4	28	7.5	2.5	50	-	12	0.25	
SABFS-M5	27	7.5	2.5	50	-	13	0.25	
SABFS-M6	26	7.5	2.5	50	-	14	0.25	

**Table 5:** Experimental conditions for determining the leachability of each radionuclide contained in different radioactive wastes (unit: weight %).

	Binder (Weight %)			Simulated Radioactive Waste (Weight %)			Water/ Binder Ratio	Radionuclide Concentration (mg/L)			Curing Method
	GGBFS (≤0.38 μm)	Alkali Reagent Ca(OH)_2_	Sulfate Reagent Ca_2_SO_4_	Concrete Waste (≥5mm)	Waste Soil (≥0.75 mm)	Metal Waste		Co	Cs	Sr	
Co	27	7.5	2.5	50	-	13	0.25	538	-	-	Air-dry
Cs	27	7.5	2.5	50	-	13	0.25	-	347	-	
Sr	27	7.5	2.5	50	-	13	0.25	-	-	487	
Co-1	31	7.5	2.5	50	9	-	0.25	21,255	-	-	Air-dry
Co-2	31	7.5	2.5	50	9	-	0.25	23,622	-	-	
Co-3	31	7.5	2.5	50	9	-	0.25	25,988	-	-	
Cs-1	31	7.5	2.5	50	9	-	0.25	-	8868	-	Air-dry
Cs-2	31	7.5	2.5	50	9	-	0.25	-	9863	-	
Cs-3	31	7.5	2.5	50	9	-	0.25	-	10,843	-	
Sr-1	31	7.5	2.5	50	9	-	0.25	-	-	15,406	Air-dry
Sr-2	31	7.5	2.5	50	9	-	0.25	-	-	17,138	
Sr-3	31	7.5	2.5	50	9	-	0.25	-	-	18,842	

**Table 6:** The chemical compositions of the coarse aggregates of concrete waste (CACW) and ground granulated blast furnace slag (GGBFS) (unit: weight %).

	SiO_2_	CaO	Al_2_O_3_	Fe_2_O_3_	K_2_O	Na_2_O_3_	MgO	SO_3_	Others	LOI
CACW (≥5 mm)	57.1	12.9	8.84	3.05	2.36	1.93	1.42	0.54	1.06	10.8
GGBFS (≤0.38 μm)	32	36.8	13.7	0.55	-	0.53	3.39	1.25	2.24	9.54

**Table 7:** The leaching indices of each radionuclide.

	Co	Cs	Sr	Co-1	Co-2	Co-3	Cs-1	Cs-2	Cs-3	Sr-1	Sr-2	Sr-3
Leachability index	14.97	14.46	7.93	22.55	16.81	8.42	19.78	13.6	7.5	15.99	14.56	6.33

## Data Availability

The data presented in this study are available on request from the corresponding author.

## Supplementary Materials

The following supporting information can be downloaded at: https://www.mdpi.com/article/10.3390/ma16196462/s1: Table S1: The standard for the self-disposal of radioactive wastes; Table S2: The standard for the radioactivity of low-level radioactive wastes. Ref. [8] is cited in the Supplementary Materials.
