# Supplementary material for: Evaluation of the Solidification of Radioactive Wastes Using Blast Furnace Slag as a Solidifying Agent
Source: Materials (Basel). 2023 Sep 28;16(19):6462. doi: 10.3390/ma16196462 (PMC10573516; doi:10.3390/ma16196462)
Supplement: Supplementary file 1 [file materials-16-06462-s001.zip › materials-2623539-supplementary.pdf]

Supplementary Materials

Table S1. The standard for the self-disposal of radioactive wastes [8].

Radioactive nuclide	Radioactivity (Bq/g)
I-129	0.01
Na-22, Sc-46, Mn-54, Co-56, Co-60, Zn-65, Nb-94, Ru-106, Ag-110m, Sb-125, Cs-134, Cs-137, Eu-152, Eu-154, Ta-182, Bi-207, Th-229, U-232, Pu-238, Pu-239, Pu-240, Pu-242, Pu-244, Am-241, Am-242m, Am-243, Cm-245, Cm-246, Cm-247, Cm-248, Cf-249, Cf-251, Es-254	0.1
C-14, Na-24, Cl-36, Sc-48, V-48, Mn-52, Fe-59, Co-57, Co-58, Se-75, Br-82, Sr-85, Sr-90, Zr-95, Nb-95, Tc-96, Tc-99, Ru-103, Ag-105, Cd-109, Sn-113, Sb-124, Te-123m, Te-132, Cs-136, Ba-140, La-140, Ce-139, Eu-155, Tb-160, Hf-181, Os-185, Ir-190, Ir-192, Tl-204, Bi-206, U-233, Np-237, Pu-236, Cm-243, Cm-244, Cf-248, Cf-250, Cf-252, Cf-254	1
Be-7, F-18, Cl-38, K-43, Ca-47, Mn-51, Mn-52m, Mn-56, Fe-52, Co-55, Co-62m, Ni-65, Zn-69m, Ga-72, As-74, As-76, Sr-91, Sr-92, Zr-93, Zr-97, Nb-93m, Nb-97, Nb-98, Mo-90, Mo-93, Mo-99, Mo-101, Tc-97, Ru-97, Ru-105, Cd-115, In-111, In-114m, Sn-125, Sb-122, Te-127m, Te-129m, Te-131m, Te-133, Te-133m, Te-134, I-126, I-130, I-131, I-132, I-133, I-134, I-135, Cs-129, Cs-132, Cs-138, Ba-131, Ce-143, Ce-144, Gd-153, W-181, W-187, Pt-191, Au-198, Hg-203, Tl-200, Tl-202, Pb-203, Po-203, Po-205, Po-207, Ra-225, Pa-230, Pa-233, U-230b, U-236, Np-240, Pu-241, Cm-242, Es-254m	10
H-3, S-35, K-42, Ca-45, Sc-47, Cr-51, Mn-53, Co-61, Ni-59, Ni-63, Cu-64, Rb-86, Sr-85m, Sr-87m, Y-91, Y-91m, Y-92, Y-93, Tc-97m, Tc-99m, Rh-105, Pd-109, Ag-111, Cd-115m, In-113m, In-115m, Te-129, Te-131, I-123, I-125,Cs-135, Ce-141, Pr-142, Nd-147, Nd-149, Sm-153, Eu-152m, Gd-159, Dy-166, Ho-166, Er-171, Tm-170, Yb-175, Lu-177, Re-188, Os-191, Os-193, Ir-194, Pt-197m, Au-199, Hg-197, Hg-197m, Tl-201, Ra-227, U-231, U-237, U-239, U-240, Np-239, Pu-234, Pu-235, Pu-237, Bk-249, Cf-253, Es-253, Fm-255	100

Table S1. *Continued.*

Radioactive nuclide	Radioactivity (Bq/g)
Si-31, P-32, P-33, Fe-55, Co-60m, Zn-69, As-73, As-77, Sr-89, Y-90, Tc-96m, Pd-103, Te-125m, Te-127, Cs-131, Cs-134m, Pr-143, Pm-147, Pm-149, Sm-151, Dy-165, Er-169, Tm-171, W-185, Re-186, Os-191m, Pt-193m, Pt-197, At-211, Th-226, Pu-243, Am-242, Cf-246	1,000
Co-58m, Ge-71, Rh-103m, Fm-254	10,000

Footnote 1: For the wastes including multiple radioactive nuclides, the criteria for their radioactivity levels are calculated as follows:

$$\sum_i \frac{C_i}{C_{L,i}} < 1$$

Where C_i is the radioactivity of the ith radionuclide and C_{Li} is the radioactivity for the self-disposal of the ith radionuclide, given in Table S2,

Footnote 2: For the radioactive nuclides excluded from Table S2, their self-disposal criteria are 0.1 Bq/g.

Table S2. The standard for the radioactivity of low-level radioactive wastes [8].

Radioactive nuclide	Radioactivity (Bq/g)
H-3	1.11E+6
C-14	2.22E+5
Co-60	3.70E+7
Ni-59	7.40E+4
Ni-63	1.11E+7
Sr-90	7.40E+4
Nb-94	1.11E+2
Tc-99	1.11E+3
I-129	3.70E+1
Cs-137	1.11E+6
Total alpha	3.70E+3
